# Case report: Immune remission from generalized myasthenia gravis in a dog with a thymoma and cholangiocellular carcinoma

**DOI:** 10.3389/fvets.2023.1124702

**Published:** 2023-03-17

**Authors:** Thomas Mignan, Robert White, Kimberley Stee, Giuseppe Bonanno, Mike Targett, Mark Lowrie

**Affiliations:** ^1^Dovecote Veterinary Hospital, CVS Group PLC, Castle Donington, United Kingdom; ^2^School of Veterinary Medicine and Science, The University of Nottingham, Sutton Bonington, United Kingdom

**Keywords:** acquired, junctionopathy, acetylcholine, thymectomy, canine

## Abstract

A 9-year-old male neutered Cockapoo was presented with an acute and progressive history of exercise induced weakness involving all limbs, and bilateral decreased ability to blink. Investigations revealed generalized myasthenia gravis alongside the presence of a thymoma and a cholangiocellular carcinoma. Symptomatic treatment through pyridostigmine bromide was used to control clinical signs, and complete surgical removal of the thymoma and cholangiocellular carcinoma was performed. Serum acetylcholine receptor antibody concentration was measured serially. Clinical remission defined as resolution of clinical signs alongside discontinuation of treatment was achieved by day 251 (8.2 months). Immune remission defined as normalization of serum acetylcholine receptor antibody concentration alongside resolution of clinical signs and discontinuation of treatment was achieved by day 566 (18.5 months). Neurological examination was normal, and the owners did not report any clinical deterioration during the final follow-up appointment on day 752 (24 months), hence outcome was considered excellent. This is the first report describing the temporal evolution of serum acetylcholine receptor antibody concentration in a dog with thymoma-associated myasthenia gravis which achieved immune remission following thymectomy. Treatment was successfully discontinued without any evidence of clinical deterioration thereafter despite serum acetylcholine receptor antibody concentration not normalizing for another 315 days (10 months).

## Introduction

Myasthenia gravis (MG) is an immune-mediated disorder impairing neuromuscular transmission through the production of antibodies against the post-synaptic component of the neuromuscular junction in skeletal muscle (1, 2). The resulting clinical presentation is skeletal muscle weakness and fatigability which can affect skeletal muscles focally or in a generalized fashion (1). In 3.4–11% of dogs with MG, the condition is associated with the presence of a cranial mediastinal mass, almost invariably a thymoma, and is thought to represent a paraneoplastic syndrome (3–6). Several other neoplasms have concurrently been reported in dogs with MG and have been suggested to also cause MG as a paraneoplastic syndrome (7–12). These include a report of a cholangiocellular carcinoma in one dog with MG (7).

In human beings, thymectomy is part of the gold standard treatment for thymoma-associated MG because it is associated with improved clinical remission rates (13). In dogs, there are currently no controlled studies evaluating the benefit of thymectomy in thymoma-associated MG, although there are reports of long term survival following thymectomy (14–17). Despite this, there is anecdotal evidence from a small number of unpublished cases that complete removal of the thymoma is associated with normalization of serum acetylcholine receptor (AChR) antibody concentration and resolution of clinical signs alongside discontinuation of treatment, collectively referred to as immune remission (14). In these cases, if the thymoma was not completely removed, serum AChR antibody concentration did not decrease, and if regrowth of the thymoma occurred, serum AChR antibody concentration would again increase thereby causing reoccurrence of clinical signs (14).

Despite this anecdotal evidence for immune remission following thymectomy in dogs with thymoma-associated MG, there are no reports of the temporal evolution of the serum AChR antibody concentration in these dogs, including time elapsed before immune remission was achieved. The latter information is currently limited to dogs with MG in the absence of concurrent neoplasia, in which immune remission is frequent, and achieved on average within 6.4 months from initial diagnosis, as well as a single case report of a dog with a thyroid carcinoma in which immune remission was achieved 5.5 months after initial presentation (11–18).

Herein we report the first case documenting the temporal evolution of serum AChR antibody concentration in thymoma-associated MG following thymectomy in which immune remission was achieved.

## Case description

A 9-year-old male neutered Cockapoo was presented to Dovecote Veterinary Hospital with an acute and progressive 4-day history of exercise induced weakness, and bilateral reduced ability to blink. Neurological examination including mentation, posture, gait, cranial nerve examination, proprioceptive testing, muscle tone and size, segmental spinal reflexes, and palpation/manipulation; was normal at rest. Upon exercise, mentation remained normal, however weakness involving all limbs, characterized by transient non-ambulatory tetraparesis and collapse was induced after ~30 s of low-grade activity. Progressive bilateral reduced ability to blink upon repeatedly performing the palpebral reflex and menace response despite normal vision was also observed, along with decreased to flaccid muscle tone to all limbs, and a decreased withdrawal reflex in all limbs. The remainder of the cranial nerve examination as well as proprioceptive testing, muscle tone and size, remaining segmental spinal reflexes, and palpation/manipulation; remained normal. The neuroanatomical localization was to a diffuse neuromuscular system disorder given the lower motor neuron clinical signs affecting all limbs upon exercise. More specifically a junctionpathy was suspected given the clinical feature of fatigability. Considered differential diagnoses were those for acute lower motor neuron tetrapesis including MG, botulism, and acute idiopathic polyradiculoneuritis. Tick paralysis was considered unlikely given the lack of travel history outside of the United Kingdom.

## Diagnostic investigations and outcome

Informed consent and contemporary standards of care were respectively obtained from the owners and maintained throughout the management of this case. Serum biochemistry and hematology profiles were unremarkable.

**Figure 1:**
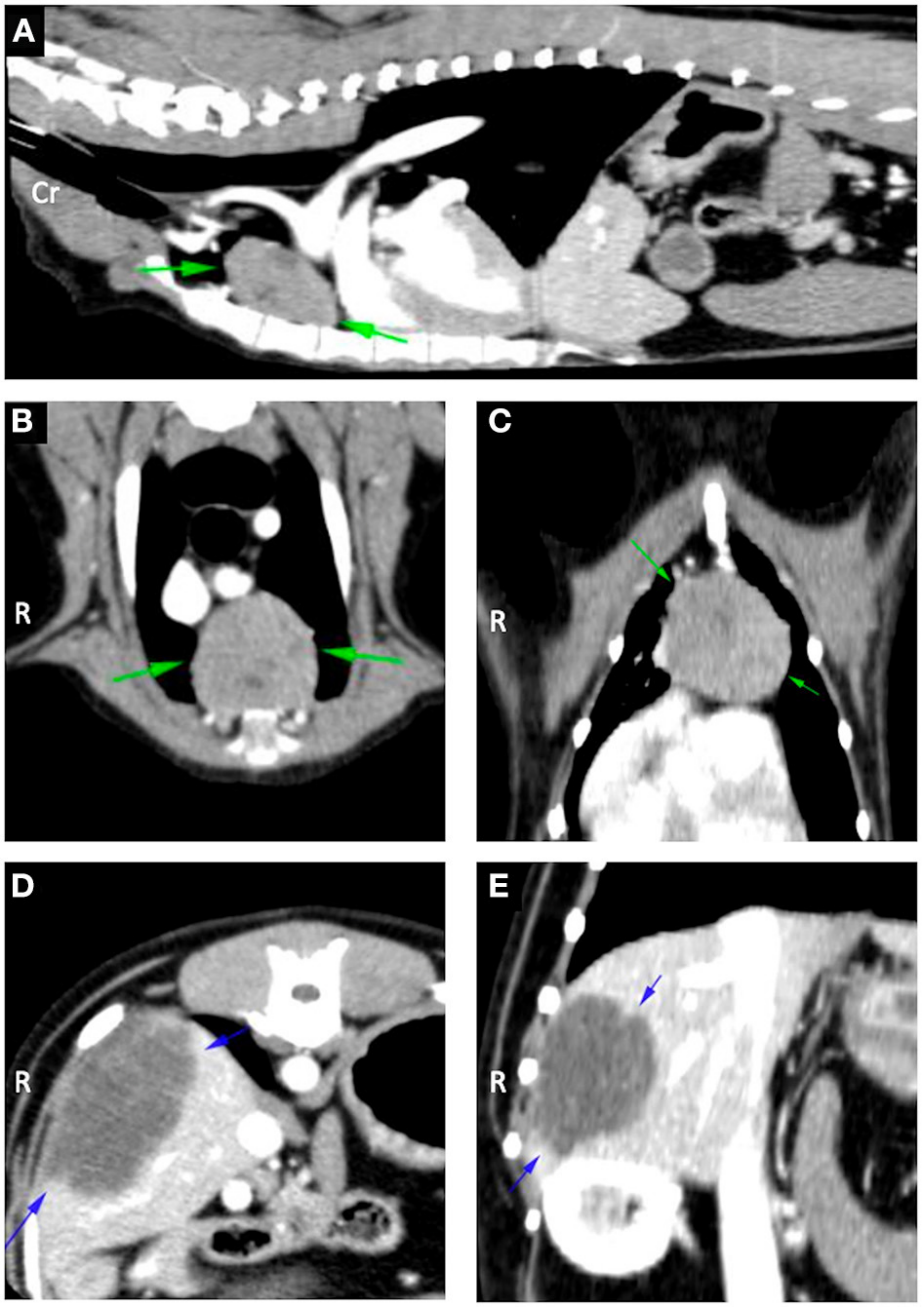
**(A)** Sagittal reconstruction contrast enhanced computed tomography image of the thorax showing the thymoma (green arrows). **(B)** Transverse contrast enhanced computed tomography image of the thorax showing the thymoma (green arrows). **(C)** Dorsal reconstruction contrast enhanced computed tomography image of the thorax showing the thymoma (green arrows). **(D)** Transverse contrast enhanced computed tomography image of the abdomen showing the cholangiocellular carcinoma (blue arrows). **(E)** Dorsal reconstruction contrast enhanced computed tomography image of the abdomen showing the cholangiocellular carcinoma (blue arrows).

Pre and post contrast computed tomography (CT) studies of the thorax and abdomen were performed under general anesthesia. A single large rounded well-defined smoothly marginated soft tissue attenuating mass was identified in the cranial mediastinum with heterogeneous contrast enhancement (Figure 1). No other abnormalities were detected upon thoracic imaging (Figure 1). Abdominal CT studies identified a single large rounded well-defined smoothly marginated hypoattenuating mass with mild contrast enhancement in the right lateral liver lobe (Figure 1). Fine needle aspiration of both masses was offered but declined by the owners.

Although electrophysiologic changes associated with denervation are not detectable for a minimum of 4–5 days after the initiating insult, investigations were pursued in this case due to the suspicion of a junctionopathy with the understanding they might need to be repeated if normal (19). Electromyography of the appendicular, axial, and head skeletal muscles was normal. Motor nerve conduction studies of the sciatic-tibial and ulnar nerves were normal bilaterally. Repetitive nerve stimulation of the sciatic-tibial, ulnar, and facial nerves on both sides of the dog at 3 Hz frequency demonstrated a decremental response (>10% decrease from the 1st to 5th stimulation) in the plantar interosseous, palmar interosseous, and orbicularis oculi skeletal muscles respectively. The absence of any abnormalities upon electromyography which are usually present with myopathies, as well as the absence of abnormalities upon motor nerve conduction studies which are usually present with motor neuropathies alongside the decremental response upon repetitive nerve stimulation which is the hallmark of impaired neuromuscular transmission; was consistent with a junctionopathy.

Serum was collected and submitted for AChR antibody concentration measurement. A provisional diagnosis of generalized myasthenia gravis was made. The cranial mediastinal mass was considered most consistent with a thymoma though other differential diagnoses were lymphoma, ectopic thyroid tissue, hematoma, or an abscess. Differential diagnoses for the liver mass were a primary neoplasia such as a hepatocellular carcinoma, hepatocellular adenoma, or a cholangiocellular carcinoma, metastatic neoplasia such as hemangiosarcoma, or a hematoma, or abscess.

Symptomatic anticholinesterase therapy with pyridostigmine bromide^1^ was started at a dosage of 0.5 mg/kg per os (PO) Q8 h on the day of initial presentation (day 0). A feeding/drinking protocol consisting of dry food/water fed/offered in an upright position that was maintained during, and, for 20 mins after completion of the meal/drink was also instituted. Within 24 h after starting pyridostigmine bromide, there was complete resolution of all clinical signs (day 1). The dog remained hospitalized for monitoring purposes for another 2 days (day 2 and 3), during which time the dog remained normal. Following this (day 4), the dog underwent a combined ventral midline sternotomy and ventral midline celiotomy and liver lobectomy procedure with surgical excision of the cranial mediastinal and liver masses in their macroscopic entirety and submission for histopathology. No neurological deterioration nor complication was observed post-operatively, and the dog was discharged from hospital 2 days later (day 6).

**Figure 2:**
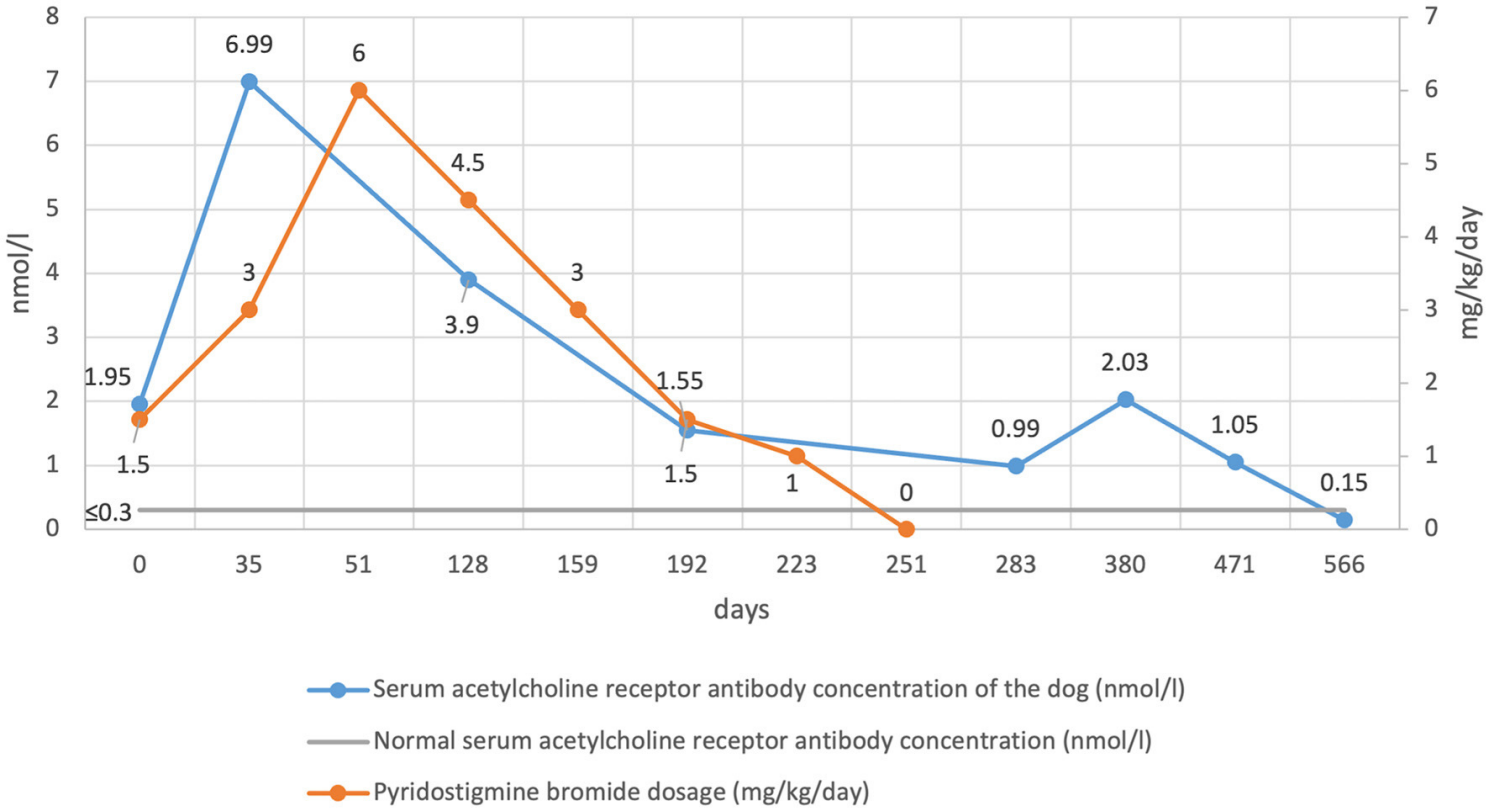
Serum acetylcholine receptor antibody concentration and pyridostigmine bromide dosage over time.

A follow-up neurological examination performed 2 weeks after discharge was normal both at rest and after exercise (day 20). At this time, the serum AChR antibody concentration result was reported as 1.95 nmol/l (day 0 concentration) (Figure 2), consistent with MG (normal < 0.6 nmol/l). Histopathology of the cranial mediastinal and liver mass revealed a diagnosis of a thymoma, and a cholangiocellular carcinoma respectively. Microscopically, both neoplasms were reported to have been completely excised. Follow-up imaging of the cholangiocellular carcinoma through contrast CT was discussed but declined by the owners.

Approximately 2 weeks later (day 35), the dog experienced a clinical deterioration characterized by re-occurrence of skeletal muscle weakness and fatigability. Measurement of serum AChR antibody concentration was repeated and had increased to 6.99 nmol/l (day 35 concentration) (Figure 2). The pyridostigmine bromide dosage was increased to 1 mg/kg PO Q8 h resulting in complete resolution of all clinical signs.

Two weeks later (day 51) the dog demonstrated another clinical deterioration. Pyridostigmine bromide dosage was increased to 1.5 mg/kg PO Q8 h, which resolved appendicular skeletal muscle weakness and fatigability, but only for 6 h post-pill, leaving a 2-h period during which clinical signs would reoccur before a dose was due (Supplementary Video 1). A further dose increase to 2 mg/kg PO Q8 h was attempted, however this resulted in adverse cholinergic effects, predominantly in the form of hypersalivation, which resolved following intravenous administration of atropine^2^ at a 0.02 mg/kg dose. A pyridostigmine bromide dosage of 1.5 mg/kg PO Q6 h was therefore attempted and resulted in sustained resolution of all clinical signs.

Over the following 2 months, the owners reported complete resolution of clinical signs. By day 128 the owners reported hypersalivation occurring 1–2 h after ingestion of pyridostigmine bromide, consistent with adverse cholinergic signs, and confirmed by direct observation in the hospital. Given this, the dosage was reduced to 1.5 mg/kg PO Q8 h, which resolved the adverse cholinergic clinical signs. Serum AChR antibody concentration was measured and was 3.9 nmol/l (day 128 concentration) (Figure 2).

Monthly re-examinations were then performed at day 159, 192 and 223. The neurological examination was normal at each assessment and the owners did not report any deterioration. Pyridostigmine bromide was gradually tapered to 1 mg/kg PO Q8 h at day 159, 0.5 mg/kg PO Q8 h at day 192 and 0.5 mg/kg PO Q12 h at day 223. Serum AChR antibody concentration on day 192 was 1.55 nmol/l. One month later (day 251), assessment remained the same and so it was decided to discontinue pyridostigmine bromide therapy. No deterioration was reported, and clinical remission defined as resolution of clinical signs alongside discontinuation of treatment was achieved.

One month later (Day 283), the clinical picture remained unchanged. Serum AChR antibody concentration was 0.99 nmol/l (Figure 2). Unsedated conscious thoracic radiographs did not reveal evidence of regrowth of the thymoma, megaoesophagus, or aspiration pneumonia. At 3-month intervals, a neurological examination was performed alongside serum AChR antibody concentration measurement (day 380, 471 and 566). The examination remained normal with a serum AChR antibody concentration of 2.03, 1.05, and 0.15 nmol/l respectively, indicating immune remission had been achieved (Figure 2).

Six months later (day 752) the neurological examination remained normal both at rest and following exercise, and no deterioration was reported by the owners, hence outcome was considered excellent.

## Discussion

This is the first report describing the temporal evolution of serum AChR antibody concentration in a dog with thymoma-associated myasthenia gravis that has achieved immune remission following thymectomy. Clinical remission, as defined as resolution of clinical signs alongside discontinuation of treatment, occurred by day 251 (8.2 months) after initial presentation. Immune remission, as defined as resolution of clinical signs alongside discontinuation of treatment and normalization of serum AChR antibody concentration, occurred by day 566 (18.5 months) after initial presentation. These times are longer than in dogs with MG in the absence of concurrent neoplasia, in which clinical and immune remission, occurred respectfully on average within 4.1 months and 6.4 months (15). In human beings with thymoma-associated MG, it is accepted that once initiated, the immune response can become self-perpetuating even after complete removal of the thymoma, due to antibody-AChR complexes in muscle-draining lymph nodes (6, 20). This might explain why immune remission occurred much later in the present case than for dogs with MG in the absence of concurrent neoplasia. Clinical and immune remission is frequent in dogs with MG in the absence of concurrent neoplasia, occurring in 88.7% of these dogs (18). However, the percentage of dogs with MG as a paraneoplastic syndrome secondary to a thymoma or another type of neoplasia that achieve immune remission is currently unknown.

Serial measurement of serum AChR antibody concentration in the absence of immunosuppression is reported to be a useful indicator of disease status in an individual animal (14). This generally held true in the present case where the increase in serum AChR antibody concentration mirrored the exacerbation of clinical signs following thymectomy, and where a decreased requirement for symptomatic therapy mirrored the decrease in serum AChR antibody concentration. However, serum AChR antibody concentration nearly doubled from 1.05 nmol/l up to 2.03 nmol/l between day 283 and day 380 without evidence of clinical deterioration, and despite treatment already having been discontinued. The cause for this asymptomatic increase in serum AChR antibody concentration is unknown and the possibility of a spurious result exists given that there are no similar reports in the human literature. Serum AChR concentration has been reported to fluctuate in some dogs with MG without a thymoma before achieving immune remission, however in these cases, the increase or decrease in serum AChR concentration mirrored clinical improvement or deterioration (4, 21).

Exacerbation of clinical signs along with an increase in serum acetylcholine receptor antibody concentration following thymectomy is frequently reported in human thymoma-associated myasthenia gravis (22). In one study, all patient with thymoma-associated MG experienced clinical deterioration and an increase in serum AChR concentration following thymectomy, questioning the overall benefit of thymectomy (22). This has also been reported in a dog with thymoma-associated myasthenia gravis, as well as in the present case (15). Seeding of AChR-like antigen in the mediastinum or activation of complement mediated AChR destruction as a direct result of the surgery have been proposed as possible theories (22). Additionally, suppressor T-cells have been found in the membrane phenotype of thymoma suggesting a possible immunomodulatory role (22).

Although the exact pathogenesis of thymoma-associated MG remains incompletely understood, it is clear that there is a relationship between the thymoma and MG in patients with thymoma-associated MG (5, 6, 14, 20). Such association is more contentious for other neoplasms. To date, thymic carcinoma, oral sarcoma, osteosarcoma, cutaneous lymphoma, cholangiocellular carcinoma, and anal sac adenocarcinoma, have concurrently been reported alongside MG in dogs (7–12). However, the majority of these are single case reports and the possibility therefore remains that these were coincidental (7–12). Furthermore, the mechanisms by which a thymoma induces MG appears related to its core role in the immune system and would therefore not occur with a neoplasia located outside of the thymus. The presence of a chollangiocellular carcinoma is believed to be incidental in this case, however its involvement alongside the thymoma in the development of MG as a paraneoplastic syndrome cannot be excluded.

It is recommended that treatment for MG be continued until serum AChR antibody concentration normalizes, however symptomatic therapy can cause severe adverse effects (14). In this case, symptomatic therapy was slowly and progressively weaned to keep the dog on the lowest effective dosage. Ultimately, treatment was discontinued and the dog remained clinically normal despite serum AChR antibody concentration not normalizing for another 315 days (10 months). This suggests that in some patients, treatment can be discontinued despite serum AChR antibody concentration being above the reference range.

The current recommended pyridostigmine bromide dosage for myasthenia gravis in dogs is 1–3 mg/kg PO Q8–12 h (23). In the present case, Q6 h administration proved necessary given that clinical signs of skeletal muscle weakness and fatigability would recur 6 h following administration of the medication, and that dose increases would cause cholinergic adverse effects. In human myasthenia gravis Q6 h and even Q4 h dosing intervals are reported, and it is recommended that not only the optimal dose but also dosage interval be determined for a given patient as was necessary in the present case (24).

Limitations of this case report include the fact that this is an isolated case preventing data analysis and extrapolation and might therefore not be representative of the population of dogs with thymoma-associated myasthenia gravis that undergo thymectomy. Furthermore, the possibility of a misdiagnosis of the liver mass cannot be ruled out given the long-term remission of the liver mass without oncological treatment whereas 88% of canine cholangiocelullar carcinomas have an aggressive behavior with common metastasis (25).

In conclusion, this is the first report describing the temporal evolution of serum AChR antibody concentration in a dog with thymoma-associated myasthenia gravis that achieved immune remission following thymectomy. Treatment was successfully discontinued without any clinical deterioration thereafter despite serum acetylcholine receptor antibody concentration not normalizing for another 315 days (10 months). Pyridostigmine bromide dosing interval was pivotal to management when dose increases were insufficient.

## Data availability statement

The original contributions presented in the study are included in the article/Supplementary material, further inquiries can be directed to the corresponding author.

## Ethics statement

Ethical review and approval was not required for this animal study because it is a retrospective case report. Written informed consent was obtained from the owners for the participation of their animal in this study.

## Author contributions

Conception and design: TM and ML. Acquisition of data, analysis and interpretation of data, revising article for intellectual content, final approval of the completed article, and drafting the article: TM, RW, KS, GB, MT, and ML. All authors contributed to the article and approved the submitted version.

## Footnotes

^1^Mestinon: Labiana pharmaceuticals, Corbera de Llobregat, Barcelona, Spain.

^2^Atropine Sulfate: Macarthys Laboratories Limited, T/A Martindale Pharma, Romford, Essex, RM3 8UG, United Kingdom.

## Supplementary material

Supplementary Video 1 Skeletal muscle weakness and fatigability occurring 6 h post-pill on day 51 when pyridostigmine bromide was administered orally at a 1.5 mg/kg Q8 h dose.
